# Translations

**Published:** 1881-11

**Authors:** Seth N. Jordan

**Affiliations:** Columbus, Ga.


### Translations.

---

BY SETH N. JORDAN, M.D., COLUMBUS, GA.

---

From Foreign Journals for the ATLANTA MEDICAL REGISTER.

---

*The Bacillus of Typhoid Fever. By E. Klebs, Arch. fur Exp. Pathologie, p.* 381.—In the course of his investigations on typhoid, Klebs comes to this result, that, in each plaque, as long as the typhoid process is in progressive development bacilli are present, so that their genetic importance is demonstrated, and the mucous membrane of the intestinal canal is to be regarded as the point of departure for all further developments of the disease. It is, also, to be presumed, that the first severe nervous symptoms are attributable to colonies of bacilli in the interstices of the pia mater. In a case with such symptoms bacilli in great numbers were found. Lobular hepatizations are likewise to be considered as resulting from a mycotic collapse of the lungs. In the beginning of the intestinal affection, the bacilli are found adherent to the mucous membrane, and can be distinguished from the numerous micro-organisms which frequently are discovered in the contenta of the intestines, by their diminutive size, and in addition they occur in rolls of thready appearance, which contain innumerable spores in their interior. Later on, the bacilli enter the tissues, notably by way of Lieberkuehn's glands, then they develop into thick tufts, which extend into the submucosa. Klebs attempted the inoculation of various animals with the bacillus typhosus in "cuturs-fluid," per os, subcutaneously, etc. The animals were variously affected. In one case the bacilli were found in the proc. vermiformis of a rabbit that died two days after the infection; and in this case the histological changes in the intestinal mucous membrane corresponded fully with the typhoid changes in the human subject. In conclusion, the author recommends the employment of large doses of the benzoates of soda or magnezia up to 20 grm =ʒvj., per die, in typhoid fever.

---

*The Uses of Resorcin. Dr. Justus Andeer, Centralblatt fur die Med. Wissenschaften.*—I. Resorcin in Diseases of the Bladder.—Andeer injected 5.0 grm.=75 grains resorcin into his own bladder and suffered little from it. In 156 cases of vesical catarrh in which this remedy was used, the results were more satisfactory than anything he had ever before employed. In catarrhs arising from gonorrhœa an injection of a 5 per cent. solution of resorcin, repeated three times, is all sufficient. In simple acute catarrh a single injection of a 1-3 per cent. sol. is all that is ever required. A chronic case of cystitis, lasting for ten years, was also cured up by a single injection of a 10 per cent. solution of resorcin. Solutions of this strength produce, however, very disagreeable tinnitus aurium.

Andeer advises that the resorcin solution be strong, at least 50 per cent., in cases of cancer of the bladder, where it can be used as an efficient disinfectant.

II. Resorcin in Cutaneous Diseases.—Resorcin is not absorbed by the healthy skin of mammals; neither does it in any concentration soever, produce irritation or paralysis when applied to the skin. Unlike chrysophanic acid, it produces no discoloration of the healthy skin. In pathological conditions it does discolor the skin, proving thereby that resorbtion has taken place. Andeer claims to have had personally the best results from resorcin in the treatment of all diseased conditions of the skin that owe their origin to living organisms. Scarlatina, erysipelas, variola, pemphigus, rupia, etc., so far as the skin affection, he has also treated satisfactorily with resorcin. Especially in burns, bites and stings of insects, wounds from infected instruments and dead bodies, Andeer claims the most brilliant results with resorcin in the form of an ointment. Andeer prefers resorcin to naphthal; firstly, because it leaves less scar when used on ulcers of the skin; secondly, it never irritates the skin, nor produces hæmoglobinuria; and for this last reason it is also to be preferred to phenol and pyrogallic acid for the same purpose.

---

*Sutures in Recent Ruptures of the Perineum. By J. Veit, Deut. Med. Wochenschrift.*—Veit advocates the immediate union of even the lesser ruptures of the perineum. To accomplish this there is need of no elaborate armamentarium—only needles and scissors are necessary. Veit recommends to begin at the perineum with the sutures; avoid deep vaginal sutures, and all superficial ones are unnecessary. After bringing the rectal mucous membrane together, the needle is passed through the perineum behind the frenulum and carried along parallel to the rupture in the vagina to the end, where it is brought through the skin. Other deep sutures can be entered under this; superficial stitches, if necessary, are placed between the deeper ones. Chloroform is only necessary in cases that are not operated on immediately post partum.

---

*Observations on the Etiology, Pathogenesis and Therapeutics of Chlorosis. By Zander, Virchow's Arch., LXXXIV, p.* 174.—To refute the opinion (not general) held by some that chlorosis is the result of an inadequate supply of iron in the food, Zander asserts that he has found iron in urine and stools of chlorotic girls, and holds that this is owing to a defective resorbtion of the iron in consequence of a faulty secretion of the digestive fluids, especially to a deficiency of hydrochloric acid. Mindful of this, he has, for a long time, treated all cases of chlorosis with hydrochloric acid: Grm. 2—4: 200.0=ʒss—ʒj : ℥6½. Sig. 1 tablespoonful after each meal. In severe cases pepsin should also be given. Zander has seen better results from this treatment than from the use of iron.

---

*Hæmatogenic Albuminuria. By Von Bamberger, Vienna Med. Wochenschrift*—Hæmatogenic albuminuria is that form, often occurring, in which it is impossible to discover any anatomical changes in the kidneys. This has also been called "transitory" albuminuria, although it can last for years. It occurs in seemingly healthy subjects, as well as in different diseases, which latter the author has divided into three classes: Febrile albuminuria, stasic albuminuria, and albuminuria appearing during violent spasm, as in epilepsy, etc. Von Bamberger believes we must look for the cause not in increased or diminished blood-pressure, but in a retardation of the circulation, and this is especially the case in febrile albuminuria. In healthy persons the cause is primarily vaso-motor influences.

---

Meynet, Rehn, and Hirschsprung have separately described, lately, cases of rheumatism, in children and adults, followed by the development of nodosities near the joints (1 case) and situate in the sheath of the tendons of the hands and feet. These nodosities were from the size of a pea to that of a hazlenut, resembling exostoses, but not hard, grouped in rows symmetrically on both sides, and disappearing spontaneously after an existence of 2–4 weeks. In one case the nodosities were found only on the forehead and on the lobes of the ears. Vulpian records several cases of gouty subjects in whom similar cartilaginous substances appeared from time to time. Two of three cases described by Hirschsprung, suffered from organic disease of the heart.

---

*Nephrectomy on Account of Uretero-Uterine Fistula. B. Crede, Arch. fur Gyn., XVII, p.* 312.—In consequence of a violent delivery, with subsequent peritonitis and exudation, patient had acquired an uretero-uterine fistula. Nephrectomy by the lumbar incision was performed, with favorable results; patient being discharged six weeks after the operation, completely cured. The extirpated kidney was shown to be diseased. Crede advises the immediate extirpation of the kidney in those cases of uretero-uterine fistula arising from septic processes in the parametrium, as the kidneys are often diseased without any symptoms being manifiest.

---

*Drainage and Drainage Tubes. By G. Neuber, Archives fur Clin. Chiurgie, XXVI, p.* 77.—Decalcinated bones have been in general use for drainage in Esmarch's clinique up to a very recent date. Not content with the irregularity of their resorbtion, Neuber was induced to seek some other method of drainage. He has reached his aim, and gives in this paper his new method, describing it as "perforation of the skin." The wound is closed up entirely and the skin is perforated at equal distances of 3-5 ctm. with an instrument resembling a shoe punch. Eighty-one operations, including 12 amputations, 13 resections, 3 osteotomies, and 6 nephrectomies, were drained in this manner, and in no case did any accidental wound complication follow, nor was there at any time a large accumulation of pus. Neuber formulates the various methods of drainage in different operations as follows: (1) In smooth, superficial incisions, as extirpation of small subcutaneous tumors, neither tubes nor perforation, but closure of the wound; (2) perforation in large wounds of the soft parts, especially such as are liable to heal in 8–10 days *per primam intentionem*, as extirpation of the mammal; (3) drainage tubes of bone or gum in wounds that heal under suppuration, as extirpation of suppurating tumors.

---

White and Harnack, in *Archives fur Exper. Pathologie*, from many experiments, conclude that tin stands of all the heavy metals next to lead, and that poisoning from tin is impossible, as taken in form of its salts it is not resorbed into the blood by way of the mucous membranes.

*Conjunctivitis Catarrhalis Acuta Intermittens. R. Hilbert, Cbl. f. Pract. Ophthalmologie.*—A day laborer, 21 years old, who lived in a house in which several persons were suffering from intermittent fever, was attacked suddenly with a conjunctivitis of both eyes; on the third day it disappeared, to return on the fifth day, in spite of the application of astringents. The spleen being considerably enlarged, with the peculiar course of the conjunctivitis, malaria was suspected, and accordingly on the sixth day one large dose of quinine was administered, which effected complete cure of the affection of the eyes.

---

*Hydrops Articulorum Intermittens. Pierson, Deut. Med. Wochenschrift.*—An eleven-year-old girl suffered for two years with swelling of the joints, which came on suddenly, and was accompanied by extraordinary painfulness. The intervals between the attacks grew gradually shorter. There existed no regular type in the appearance of the puffiness of the joints, and the affection attacked almost every joint. Galvanization of the cervical vertebræ, in conjunction with the administration of arsenic, exercised a beneficial influence on the trouble.

---

Ulcus varicosum is treated by Meyerhoff, in *Deut. Med. Wochenscrift*, with subcutaneous injections of secale cornutum. 1 centigram$=\frac{1}{6}$ gr. is injected between the reticulated veins every other day, 8–10 times, then bandage the leg during the day and command rest. Pain lasts 2–6 hours. Eczema with vascularization, is successfully treated in the same manner.

---

Hindenlang, in *Berlin Klin. Wochenschrift*, recommends metaphosphoric acid as a safe and efficient reagent for albumen. Immediately before use dissolve a small piece in water, and add this to the urine to be examined. A cloudy solution appears when only the slightest traces of albumen are present.

---

Methyl blue, according to Ehrlich, in *Zeitschrift f. Klin. Med.*, is the most appropriate coloring material for exhibiting micro-organisms in the blood.

Talamon, in *Progrès Med.*, claims to have found in eight cases of diphtheria a mycelium, load, linked and the size of a red-blood corpuscle. He produced pseudo-membranes in the throat of pigeons by inoculating the spores in "cuturs-fluid."

---

Peyrusson, in *Complesrendus*, recommends a mixture of four parts each of alcohol 90$^{\circ}$ and nitric acid of 1.33 spec. gravity to be used in the place of nitrite of ethyl as a disinfectant.

